# Prognostic factors for rural endometrial cancer patients in a population-based cohort

**DOI:** 10.1186/s12889-019-7262-7

**Published:** 2019-07-10

**Authors:** Brenna E. Blackburn, Sean Soisson, Kerry Rowe, John Snyder, Alison Fraser, Vikrant Deshmukh, Michael Newman, Ken Smith, Kimberly Herget, Anne C. Kirchhoff, Deanna Kepka, Theresa L. Werner, David Gaffney, Kathi Mooney, Mia Hashibe

**Affiliations:** 1 Division of Public Health, Department of Family and Preventive Medicine, Huntsman Cancer Institute, University of Utah School of Medicine, 2000 Circle of Hope, Salt Lake City, UT 84112 USA; 2 Huntsman Cancer Institute, Salt Lake City, UT USA; 3 Intermountain Healthcare, Salt Lake City, UT USA; 4 Pedigree and Population Resource, Population Sciences, Huntsman Cancer Institute, Salt Lake City, UT USA; 5 University of Utah Health Sciences Center, Salt Lake City, UT USA; 6 Utah Cancer Registry, University of Utah, Salt Lake City, UT USA; 7 Department of Pediatrics, University of Utah School of Medicine, Salt Lake City, UT USA; 8 Department of Pediatrics, University of Utah School of Medicine, Salt Lake City, UT USA; 9 Division of Oncology, Department of Medicine, University of Utah School of Medicine, Salt Lake City, UT USA; 10 Department of Radiation Oncology, University of Utah School of Medicine, Salt Lake City, UT USA; 11 College of Nursing, University of Utah, Salt Lake City, UT USA

## Abstract

**Background:**

Endometrial cancer is the second most common cancer among female cancer survivors in the US and is increasing in incidence. Rural endometrial cancer patients experience lower survival rates but the reasons for the lower survival are not known. The aim of this study is to examine whether prognostic factors are different for rural and urban patients in a population-based cohort.

**Methods:**

Endometrial cancer patients diagnosed 1997-2012 were identified through the Utah Cancer Registry and Utah Population Database. The address at cancer diagnosis was used to classify patients in rural or urban residences. Demographic and cancer-specific characteristics were examined as prognostic factors for both all-cause and endometrial cancer-specific mortality using Cox proportional hazards models.

**Results:**

There were 2,994 endometrial cancer patients and 14.1% of these patients lived in rural areas at diagnosis. Rural endometrial cancer patients were older at cancer diagnosis and did not appear to be different in terms of obesity or overweight at cancer diagnosis. There were no differences for treatment or stage at diagnosis although rural patients had higher proportions of higher grade. Age at diagnosis, poverty, education, and histology were significant prognostic factors for all-cause death. Rural patients with more advanced stages of cancer had significantly increased risks of all-cause and endometrial cancer-specific death than urban patients. Rural endometrial cancer patients diagnosed at advanced stage had a 17-fold increase in the risk of all-cause death compared to an 8-fold increase in death for urban patients.

**Conclusions:**

Rural endometrial cancer patients in Utah were older at diagnosis, had higher grade and higher comorbidities. While urban and rural endometrial cancer patients shared many prognostic factors, the risk of mortality is greater among rural patients with advanced stage endometrial cancer. Future studies should examine where patients are receiving treatment and how that impacts their survival and how to reduce the mortality rates of high risk patients.

## Introduction

Endometrial cancer is the second most common cancer among female cancer survivors in the US, with an estimated 757,190 survivors in 2017 [1]. In addition, it is one of the few cancers in the US with an increasing incidence rates [2]. The death rate for this cancer has also been increasing, with an average increase of 1.4% per year between 2005-2014 and an estimated 10,470 deaths in 2016 [3, 4]. The overall five-year survival for endometrial cancer is 87% [3].

Older age, higher stage, grade, race, comorbidities, obesity, and treatment are associated with lower endometrial cancer survival [5–8]. There have been conflicting results in the associations between cancer survival and rural residence [5, 7, 9]. Bregar et al. reported decreased overall mortality for higher stage endometrial cancer patients in rural communities with no significant difference in lower stage patients in a population of more than 42,000 patients [7]. While Modesitt et al. reported significantly increased disease-specific survival for patients in rural areas, there was no significant difference in overall survival among 3,562 endometrial cancer patients [9].

Endometrial cancer treatment can include surgery, chemotherapy, radiation, hormone therapy, and/or targeted therapy [10]. Many of these treatment options require multiple visits over a longer period of time. Rural patients may have a greater geographic accessibility burden, which could account for the differences in treatment have been observed between rural and urban endometrial cancer patients [9, 11]. Rural patients received a less comprehensive surgical evaluation and are less likely to have multimodality treatment and have any lymph nodes removed [9, 11]. These treatment differences have been previously associated with lower survival rural endometrial cancer patients [11]. While previous studies have examined treatment differences as a factor in survival differences, they have not examined how prognostic factors, such as age at diagnosis, baseline health, and stage of diagnosis may be associated with decreased survival in rural areas. The aim of this study is to examine whether prognostic factors are different for rural and urban patients, as well as to examine the trends of treatment and mortality over time in rural and urban areas. We examined prognostic factors in a statewide population-based cohort of first primary endometrial cancer patients who were linked to medical record data, cancer registry data, death records, and demographic data from the Utah Department of Health.

## Methods

This cohort was established within the Utah Population Database (UPDB), which links data from the Utah Cancer Registry (UCR) (one of the original NCI SEER cancer registries), electronic medical records (EMR), statewide healthcare data, voter registration records, residential histories, family history records, and birth and death certificates [12]. The healthcare data from UPDB includes ambulatory surgery and inpatient discharge data from the entire state of Utah as well as linkage to EMR data from 2 of the biggest healthcare providers in Utah, the University of Utah Healthcare and Intermountain Healthcare.

First primary endometrial cancer cases diagnosed between 1997 and 2012 were identified through UCR (SEER ICD-O-3 codes: C54.0-C55.9). Death dates were captured locally using Death certificates as well as nationwide genealogy, the Social Security Death Index (nationwide), and UCR records. Participants with endometrial cancer were excluded if the cancer was in situ (n=183), the cancer stage was unknown/missing (n=153), or if grade was missing (n=471). Follow up time was calculated as time from cancer diagnosis to either death or to their last known date to be alive and residing in Utah.

All participants were linked to the available healthcare data in the UPDB. International Classification of Diseases, Ninth Revision, Clinical Modification (ICD-9-CM) codes prior to cancer diagnosis were used to create the Charlson Comorbidity Index (CCI) for each patient at the time of cancer diagnosis [13]. Cause of death codes (ICD-10) were used to classify deaths as all-cause and endometrial cancer specific deaths (C541, C549, C55). Residence at cancer diagnosis was collected through several sources in the UPDB. The mean time from diagnosis to the date the residence was captured was 0.44 years. The zip codes were linked to the Rural Urban Commuting Area Codes (RUCA) Version 2.0 and each zip code was designated as either urban or rural based on the RUCA level [14]. This was based on the metropolitan/nonmetropolitan definition where all zip codes within an urbanized area core (population > 50,000) and those zip codes with more than 25% of their population commuting to urbanized area core [15]. All zip codes were also linked to poverty and education data obtained through UDS Mapper, which incorporates data from the American Community Survey [16]. The poverty data used were the percentage of population in each zip code below the federal poverty level. The education data were the percentage of population in each zip code who had not obtained a high school diploma.

Endometrial cancer histologies were categorized using SEER ICD-O-3 morphology codes. Cancers were classified as type I and II based on histological subtypes: adenocarcinoma, endometrioid, mucinous adenocarcinoma, and adenocarcinoma with squamous differentiation were classified as type I (ICD-O-3 morphology codes: 8140, 8260, 8380, 8382, 8480, 8482, 8560, and 8570) and clear-cell carcinomas and papillary serous carcinomas as type II (ICD-O-3 morphology codes: 8310, 8441, and 8460) which were grade 3 or higher [17, 18].

### Statistical Methods

The earliest body mass index (BMI) measurement at least 1 year before cancer diagnosis was calculated to assess baseline BMI. Approximately 28% of all subjects were missing BMI, thus we imputed BMI for the 28% who were missing it using age at diagnosis, sex, race, and CCI as predictors using multiple imputation. We compared Cox proportional hazards regression models including only those who had BMI in the data and with the full study population, including those who had imputed BMI, to assure that our inferences did not change due to the imputed BMI.

Chi-square tests were used to assess differences in the demographic characteristics between endometrial cancer patients in rural and urban areas. Unadjusted Kaplan-Meier survival curves by residence were created and the logrank test was used to compare the survival between rural and urban endometrial cancer patients. Cox proportional hazards were used to calculate hazard ratios for the risk factors of all-cause death as well as endometrial cancer specific death. Models were adjusted on potential confounders selected based on prior knowledge as appropriate. All models were stratified by residence (urban/rural). All analyses were conducted using SAS 9.4. This study was approved by the University of Utah Institutional Review Board.

## Results

**Fig. 1:**
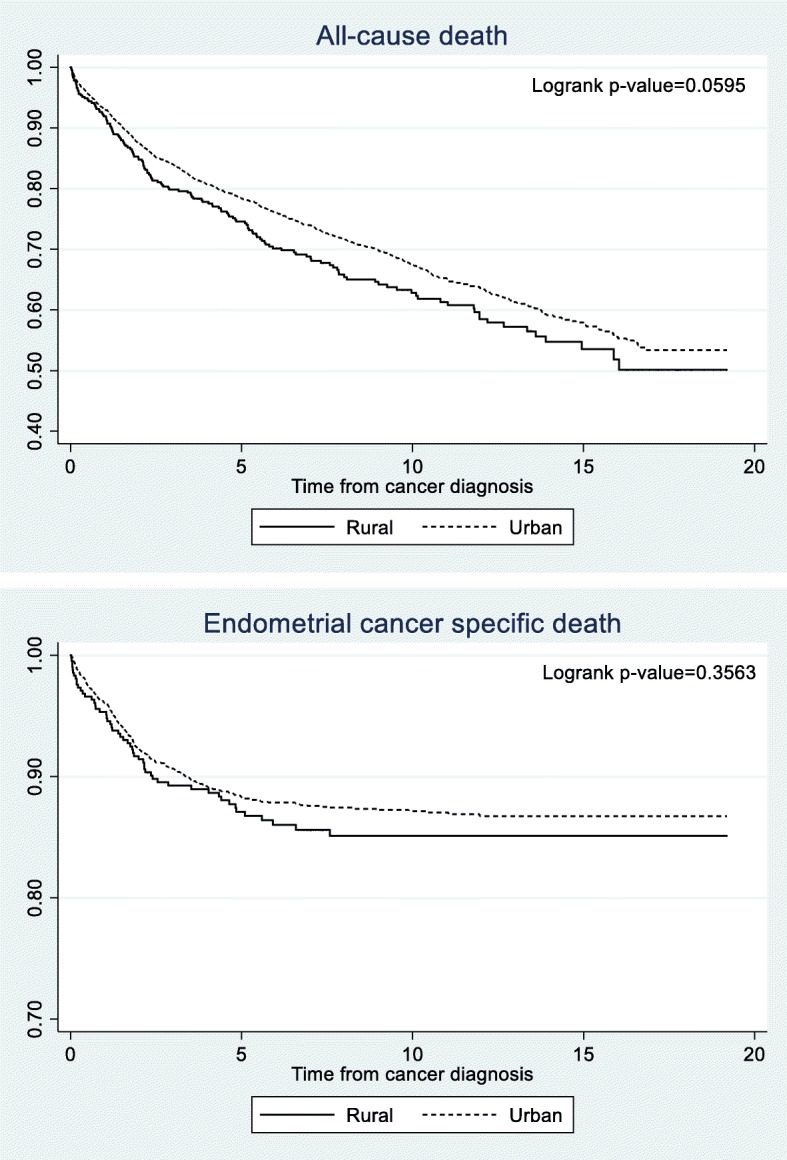
Unadjusted Kaplan-Meier survival curves for all-cause death and endometrial cancer specific death by residence (rural/urban) with logrank test *p*-value

There were a total of 2,994 endometrial cancer patients included in this study, with 85% (2,573) living in urban areas at diagnosis. The average follow-up time was 7.3 years (standard deviation=6.2). The majority of patients were overweight (29.1%) or obese (43.4%) at diagnosis. Patients in urban areas were significantly younger on average at diagnosis (60.8 vs 63.0 years old, *P = .0009*) and had significantly lower mortality (*P = .0246*) than those in rural areas (Table 1). Table 2 shows the clinical characteristics by residence. The majority of patients in both rural (67.0%) and urban (65.2%) areas received surgery as their only treatment for the endometrial cancer. Grade of endometrial cancer was significantly different between the 2 groups with the urban population having higher rates of grade I (48.2% vs 43.5%). Figure 1 shows the survival curves for all-cause death and endometrial cancer specific death by residence. While in both survival curves, rural endometrial cancer patients had lower survival, neither all-cause death nor endometrial cancer specific death had a significant *p-*value in the logrank test.

**Table 1:** Demographic characteristics of endometrial cancer patients by residence at diagnosis

	Total	Urban	Rural	*p*-value
	N=2,994	N=2,573	N=421	
	N (%)	N(%)	N(%)	
Age at cancer diagnosis				
< 40	162 (5.4)	147 (5.7)	15 (3.6)	0.0068
40-49	349 (11.7)	303 (11.8)	46 (10.9)	
50-59	862 (28.8)	757 (29.4)	105 (24.9)	
60-69	845 (28.2)	729 (28.3)	116 (27.6)	
70-79	522 (17.4)	425 (16.5)	97 (23.0)	
80+	254 (8.5)	212 (8.2)	42 (10.0)	
Diagnosis year				
1997-2000	637 (21.3)	541 (21.0)	96 (22.8)	0.5528
2001-2004	695 (23.2)	591 (23.0)	104 (24.7)	
2005-2008	773 (25.8)	674 (26.2)	99 (23.5)	
2009-2012	889 (29.7)	767 (29.8)	122 (29.0)	
Body mass index at diagnosis				
<18 kg/m^2^	31 (1.0)	26 (1.0)	5 (1.2)	0.7549
18-24.9 kg/m^2^	795 (26.6)	688 (26.7)	107 (25.4)	
25-29.9 kg/m^2^	870 (29.1)	753 (29.3)	117 (27.8)	
30+ kg/m^2^	1,298 (43.4)	1106 (43.0)	192 (45.6)	
Race				
White	2,847 (95.1)	2,446 (95.1)	401 (95.3)	0.8028
Other	134 (4.5)	115 (4.5)	9 (4.5)	
Missing	13 (0.4)	12 (0.5)	1 (0.2)	
Charlson Comorbdity Index				
0	1,830 (61.1)	1561 (60.7)	269 (63.9)	0.0467
1	640 (21.4)	569 (22.1)	71 (16.9)	
2+	524 (17.5)	443 (17.2)	81 (19.2)	
Vital Status				
Alive	2,061 (68.8)	1,791 (69.6)	270 (64.1)	0.0246
Dead	933 (31.2)	782 (30.4)	151 (35.9)	
Cause of death				
Endometrial cancer COD	335 (11.9)	284 (11.0)	51 (12.1)	0.5160
Cancer (not endometrial) COD	520 (17.4)	441 (17.1)	79 (18.8)	0.4145
Non cancer COD	318 (10.6)	263 (10.2)	55 (13.1)	0.0793
Family history of any cancer				
First degree relative	1,149 (38.4)	955 (37.1)	194 (46.1)	0.0005
Second degree relative	1,362 (45.5)	1,132 (44.0)	230 (54.6)	<0.0001
Third degree relative	1,316 (44.0)	1,087 (42.3)	229 (54.4)	<0.0001
Any relative	1,654 (55.2)	1,383 (53.8)	271 (64.4)	<0.0001
Family history of endometrial cancer				
First degree relative	116 (3.9)	89 (3.5)	27 (6.4)	0.0036
Second degree relative	184 (6.2)	156 (6.1)	28 (6.7)	0.6415
Third degree relative	259 (8.7)	214 (8.3)	45 (10.7)	0.1085
Any relative	494 (16.5)	404 (15.7)	90 (21.4)	0.0036
	Mean (std)	Mean (std)	Mean (std)	p-value
Percent of zip code living below poverty	12.7% (7.1%)	12.5% (7.0%)	14.2% (7.4%)	<0.0001
Percent of zip code without a high school diploma	9.1% (6.6%)	8.8% (6.7%)	10.4% (5.9%)	<0.0001

**Table 2:** Clinical characteristics of endometrial cancer patients by residence at diagnosis

	Total	Urban	Rural	p-value
	N=2,994	N=2,573	N=421	
	N (%)	N(%)	N(%)	
Treatment				
Surgery only	1,961 (65.5)	1,679 (65.2)	282 (67.0)	0.1452
Surgery and radiation	605 (20.2)	537 (20.9)	68 (16.2)	
Surgery, radiation, and chemotherapy	131 (4.4)	110 (4.3)	21 (5.0)	
Other combination	130 (4.3)	112 (4.4)	18 (4.3)	
None	45 (1.5)	36 (1.4)	9 (2.1)	
Missing	122 (4.1)	99 (3.9)	23 (5.5)	
Grade				
Grade I (Well differentiated)	1,422 (47.5)	1,239 (48.2)	183 (43.5)	0.0194
Grade II (Moderately differentiated)	921 (30.8)	783 (30.4)	138 (32.8)	
Grade III (Poorly differentiated)	549 (18.3)	473 (18.4)	76 (18.1)	
Grade IV (Undifferentiated)	102 (3.4)	78 (3.0)	24 (5.7)	
Cancer stage at diagnosis				
Local (Stage I)	2,304 (77.0)	1,993 (77.5)	311 (73.9)	0.2383
Regional (Stage II)	521 (17.4)	436 (17.0)	85 (20.2)	
Advanced (Stage III and IV)	169 (5.6)	144 (5.6)	25 (5.9)	
Type				
Type I	2,220 (74.2)	1,918 (74.5)	302 (71.7)	0.2697
Type II	430 (14.4)	369 (14.3)	61 (14.5)	
Other	344 (11.5)	286 (11.1)	58 (13.8)	
Histology				
Endometrioid adenocarcinoma	2,035 (68.0)	1,779 (69.1)	256 (60.8)	0.0649
Adenocarcinoma, NOS	388 (13.0)	314 (12.2)	74 (17.6)	
Adenocarcinoma with squamous differentiation	63 (2.1)	57 (2.2)	6 (1.4)	
Serous adenocarcinoma	105 (3.5)	90 (3.5)	15 (3.6)	
Clear cell carcinoma	24 (0.8)	19 (0.7)	5 (1.2)	
Mixed cell adenocarcinoma	55 (1.8)	45 (1.8)	10 (2.4)	
Mucinous adenocarcinoma	49 (1.6)	41 (1.6)	8 (1.9)	
Carcinosarcoma	45 (1.5)	38 (1.5)	7 (1.7)	
Stromal sarcoma	52 (1.7)	44 (1.7)	8 (1.9)	
Leiomyosarcoma	51 (1.7)	43 (1.7)	8 (1.9)	
Other	127 (4.2)	103 (4.0)	24 (5.7)

While the crude hazard ratio for all-cause death was significantly increased for rural patients compared to urban patients, increased risks were not observed in the adjusted models for both all-cause death (HR=1.12, 95% CI=0.94, 1.35) and endometrial cancer specific death (HR=1.09, 95% CI=0.80, 1.48). All-cause risk of death significantly decreased in 2005-2008 and 2009-2012 in urban endometrial patients (HR=0.70, 95% CI=0.56, 0.86 and HR=0.74, 95% CI=0.59, 0.93) (Table 3). However, this decrease was not observed among rural endometrial cancer patients (HR=1.29, 95% CI=0.81, 2.07 and HR=1.32, 95% CI=0.81, 2.14). Neither urban nor rural residence had significant changes in endometrial cancer specific death over time. The risk of all-cause death and death from endometrial cancer were lower in nearly all age groups in urban areas than in rural areas. However, there was no significant difference between rural vs. urban residence. Neither BMI nor CCI were associated with an increased risk for all-cause death or endometrial cancer death. However, urban patients with a CCI score of 2 or greater had significantly lower rates of endometrial cancer death (HR=0.70, 95% CI=0.52, 0.94).

**Table 3:** Hazard ratios for survival by demographic characteristics by residence at diagnosis

	All Death		Endometrial Cancer Death	
	Urban	Rural	Urban	Rural
	Adjusted HR (95%CI)	Adjusted HR (95%CI)	Adjusted HR (95%CI)	Adjusted HR (95%CI)
Age at cancer diagnosis^a^				
< 40	0.28 (0.16, 0.48)	0.79 (0.19, 3.41)	0.43 (0.22, 0.85)	--
40-49	0.36 (0.26, 0.52)	0.64 (0.28, 1.47)	0.46 (0.27, 0.79)	0.26 (0.03, 2.06)
50-59	0.63 (0.50, 0.79)	0.85 (0.48, 1.51)	0.75 (0.53, 1.07)	1.09 (0.44, 2.68)
60-69	Reference	Reference	Reference	Reference
70-79	2.02 (1.67, 2.45)	2.73 (1.74, 4.28)	1.73 (1.26, 2.36)	2.49 (1.16, 5.34)
80+	3.95 (3.19, 4.88)	6.28 (3.76, 10.48)	2.51 (1.70, 3.71)	4.36 (1.79, 10.63)
Diagnosis year^b^				
1997-2000	Reference	Reference	Reference	Reference
2001-2004	0.86 (0.72, 1.04)	0.88 (0.56, 1.38)	1.11 (0.79, 1.57)	1.74 (0.72, 4.23)
2005- 2008	0.70 (0.56, 0.86)	1.29 (0.81, 2.07)*	0.94 (0.66, 1.35)	2.35 (0.99, 5.56)
2009-2012	0.74 (0.59, 0.93)	1.32 (0.81, 2.14)*	1.16 (0.82, 1.65)	1.85 (0.76, 4.50)
Body mass index at baseline^c^				
<18 kg/m^2^	1.31 (0.67, 2.56)	1.29 (0.31, 5.38)	1.41 (0.52, 3.87)	4.11 (0.91, 18.53)
18-24.9 kg/m^2^	Reference	Reference	Reference	Reference
25-29.9 kg/m^2^	0.92 (0.77, 1.11)	1.08 (0.69, 1.69)	0.92 (0.68, 1.25)	0.67 (0.29, 1.53)
30+ kg/m^2^	1.03 (0.87, 1.23)	1.38 (0.91, 2.10)	0.99 (0.74, 1.33)	1.39 (0.69, 2.78)
Race^d^				
White	Reference	Reference	Reference	Reference
Non-White	1.02 (0.71, 1.47)	0.43 (0.14, 1.35)	1.69 (1.06, 2.69)	0.86 (0.21, 3.54)
Charlson Comorbdity Index^e^				
0	Reference	Reference	Reference	Reference
1	1.08 (0.86, 1.36)	1.17 (0.70, 1.97)	0.86 (0.62, 1.19)	1.02 (0.46, 2.26)
2+	1.20 (0.98, 1.46)	1.09 (0.70, 1.69)	0.70 (0.52, 0.94)	0.72 (0.36, 1.46)
Percent of people in their zip code living below poverty^f^				
≤11.3%	Reference	Reference	Reference	Reference
>11.3%	1.38 (1.19, 1.59)	1.39 (1.00, 1.94)	1.18 (0.93, 1.50)	1.22 (0.69, 2.15)
Percent of people in their zip code without a high school diploma^f^				
≤7.3%	Reference	Reference	Reference	Reference
>7.3%	1.28 (1.11, 1.47)	0.95 (0.66, 1.36)	1.18 (0.94, 1.50)	0.88 (0.48, 1.64)

* Hazard Ratios between residence groups *p*-value <0.05

^a^ adjusted for year of diagnosis, BMI, CCI, race, stage, grade, education, and poverty

^b^ adjusted for BMI, CCI, and age at diagnosis

^c^ adjusted for age at diagnosis, race, education, and poverty

^d^ not adjusted

^e^ adjusted for age at diagnosis, race, education, and poverty

^f^ adjusted for age at diagnosis and race

Table 4 shows the hazard ratios for clinical characteristics by residence for both all-cause death and endometrial cancer specific death. Both treatment and tumor type were significantly associated with all-cause death and endometrial cancer specific death in both urban and rural areas. Patients with regional and advanced endometrial cancer when compared to local endometrial cancer had significantly increased risks in both all-cause death and endometrial cancer specific deaths in rural areas compared to urban areas. The risks for endometrial cancer specific death were more than doubled in rural areas compared to urban areas for regional cancer (HR=7.63, 95% CI=3.64, 16.01 vs HR=3.08, 95% CI=2.31, 4.10). Two histologies were associated with significantly higher risk of all-cause death in rural patients: serous adenocarcinoma (HR=2.16, 95% CI=1.05, 4.48 vs HR=0.90, 95% CI=0.66, 1.24) and clear cell carcinoma (HR=6.45, 95% CI=2.37, 17.54 vs HR=1.84, 95% CI=1.00, 3.38). Those with clear cell carcinoma also had a significantly increased risk in rural areas compared to urban areas for endometrial cancer specific death (HR=10.85, 95% CI=3.12, 37.73 vs HR=0.58, 95% CI=0.14, 2.34).

**Table 4:** Hazard ratios for survival by clinical characteristics by residence at diagnosis

	All Death		Endometrial Cancer Death	
	Urban	Rural	Urban	Rural
	Adjusted HR (95%CI)	Adjusted HR (95%CI)	Adjusted HR (95%CI)	Adjusted HR (95%CI)
Treatment^a^				
Surgery only	Reference	Reference	Reference	Reference
Surgery and radiation	1.44 (1.19, 1.73)	1.31 (0.80, 2.14)	1.99 (1.49, 2.66)	2.10 (0.97, 4.56)
Surgery, radiation, and chemotherapy	2.85 (1.99, 4.09)	2.17 (0.97, 4.84)	3.81 (2.41, 6.04)	3.52 (1.12, 11.07)
Other combination	7.23 (5.53, 9.45)	4.01 (1.95, 8.28)	10.29 (7.28, 15.54)	7.21 (2.64, 19.70)
Grade^b^				
Grade I (Well differentiated)	Reference	Reference	Reference	Reference
Grade II (Moderately differentiated)	1.53 (1.27, 1.84)	1.11 (0.73, 1.70)	4.01 (2.57, 6.27)	1.27 (0.50, 3.23)*
Grade III (Poorly differentiated)	2.81 (2.31, 3.43)	2.60 (1.70, 3.97)	10.74 (6.94, 16.62)	5.31 (2.37, 11.92)
Grade IV (Undifferentiated)	4.16 (3.00, 5.77)	8.54 (4.63, 15.77)*	15.46 (9.02, 26.50)	13.51 (5.03, 36.31)
Cancer stage at diagnosis^c^				
Local (Stage I)	Reference	Reference	Reference	Reference
Regional (Stage II)	1.95 (1.64, 2.33)	3.09 (2.08, 4.59)*	3.08 (2.31, 4.10)	7.63 (3.64, 16.01)*
Advanced (Stage III and IV)	7.98 (6.34, 10.04)	16.54 (9.45, 28.96)*	10.91 (7.88, 15.12)	42.87 (17.28, 106.34)*
Type^d^				
Type I	Reference	Reference	Reference	Reference
Type II	2.72 (2.27, 3.24)	3.24 (2.12, 4.96)	5.68 (4.27, 7.57)	8.42 (4.16, 17.05)
Other	3.57 (2.98, 4.27)	3.61 (2.41, 5.41)	8.44 (6.33, 11.25)	7.46 (3.64, 15.29)
Histology^e^				
Endometrioid adenocarcinoma	Reference	Reference	Reference	Reference
Adenocarcinoma, NOS	0.98 (0.78, 1.22)	1.10 (0.66, 1.86)	0.74 (0.47, 1.19)	0.44 (0.09, 2.04)
Adenocarcinoma with squamous differentiation	1.26 (0.80, 1.99)	1.28 (0.43, 3.81)	1.25 (0.60, 2.60)	--
Serous adenocarcinoma	0.90 (0.66, 1.24)	2.16 (1.05, 4.48)*	0.81 (0.52, 1.27)	2.50 (0.83, 7.51)
Clear cell carcinoma	1.84 (1.00, 3.38)	6.45 (2.37, 17.54)*	0.58 (0.14, 2.34)	10.85 (3.12, 37.73)*
Mixed cell adenocarcinoma	1.12 (0.65, 1.94)	0.98 (0.29, 3.27)	0.59 (0.24, 1.46)	0.67 (0.08, 5.65)
Mucinous adenocarcinoma	0.94 (0.57, 1.56)	0.38 (0.05, 2.75)	1.19 (0.48, 2.93)	--
Carcinosarcoma	1.89 (1.28, 2.78)	1.00 (0.39, 2.57)	2.40 (1.46, 3.92)	0.75 (0.18, 3.21)
Stromal sarcoma	1.81 (1.09, 3.01)	0.56 (0.15, 2.08)	1.25 (0.59, 2.62)	1.19 (0.23, 6.27)
Leiomyosarcoma	2.00 (1.32, 3.03)	3.75 (1.45, 9.73)	1.16 (0.64, 2.10)	1.77 (0.34, 9.18)
Other	1.69 (1.26, 2.25)	1.73 (0.95, 3.16)	1.35 (0.87, 2.10)	2.05 (0.82, 5.16)

* Hazard Ratios between residence groups *p*-value <0.05

^a^ adjusted for BMI, race, year of diagnosis, age at diagnosis, CCI, poverty, and education

^b^ adjusted for stage, race, age at diagnosis, year of diagnosis, BMI, and CCI

^c^ adjusted for grade, race, age at diagnosis, year of diagnosis, BMI, CCI, poverty, and education

^d^ adjusted for BMI, race, age at diagnosis, CCI, poverty, and education

^e^ adjusted for age at diagnosis, year of diagnosis, BMI, race, CCI, grade, and stage

## Discussion

We conducted the first population-based assessment of prognostic factors in urban and rural areas among endometrial cancer patients. As expected, age at cancer diagnosis, treatment, grade, stage, type, and histology were significant risk factors for all-cause death. However, the levels of risk were different between urban and rural endometrial cancer patients in Utah. Endometrial cancer patients with regional and advanced stages disease were significantly more likely to die either due to all-cause and endometrial cancer specific mortality in rural areas than in urban areas. There were also differences in histology and grade, with rural endometrial cancer patients with clear cell carcinoma having significantly increased risk of death and urban endometrial cancer patients with higher grade cancer having increased risk of death.

Studies have shown that rural cancer survivors have worse overall health than urban cancer survivors [19]. This includes comorbidities, psychological distress, and lower levels of physical activity [19, 20]. A recent study observed significantly increased risk of death in rural endometrial cancer patients compared to urban patients, however they were not able to include BMI and baseline health which we included [11]. Generally, in our study rural endometrial cancer patients had higher rates of multiple comorbidities. Much of these differences have been explained through socioeconomic status (SES) and education [9]. While those living in rural areas in our study overall lived in zip codes with higher rates of poverty and lower rates of education, we did not observe any significant differences of the effect of education and poverty on mortality between urban and rural patients.

We observed that patients in rural areas with regional and advanced endometrial cancer had significantly higher risk of both endometrial cancer specific death and all-cause death than those in urban areas. This may be a result of reduced access to healthcare. Patients in rural areas are likely to live much farther from a cancer center and may have less access to adjuvant therapy, which has been associated with decreased survival [11, 21]. Patients may also be offered therapy, but may decline it due to reasons such as distance and cost. This difference in treatment, especially treatment beyond surgery, may account for decreased survival in rural areas.

Non-white endometrial cancer patients living in urban areas had a significantly increased risk for endometrial cancer specific death. This risk was not increased for rural endometrial cancer patients, which may be due to the small sample of non-white patients (n=19). Decreased survival has been well established in black women with endometrial cancer when compared to white women [22–24]. Out of the non-white urban population in our study, only 12 (10.4%) were black. The other non-white women were Asian and Pacific Islander (66.1%) and American Indian (23.5%). Asians have similar or higher survival rates to non-Hispanic white women, whereas American Indian/Alaska Natives h have worse survival [23, 25]. The sample sizes of individual non-white racial groups were too small to determine alone.

This study has several limitations. First, the population includes only to endometrial cancer patients diagnosed in Utah. Utah has a less diverse population than the rest of the nation on average and tends to be one the healthiest states. This allowed for a more homogenous study population. Another limitation is having a small rural population. There were several risk factors where we may not have had enough power to detect a significant risk, as well as risk factors, like some histologies, that did not have any rural patients. We also did not have data on where the patients received treatment, only where their residence was at the time of diagnosis. Patients in rural areas may have travelled to large cancer centers for treatment or have been offered treatment and had to decline due to distance, which we could not assess.

There are several strengths to this study as well. This is a statewide study covering a time period of more than 15 years. The major strength of this study is the population based design with nearly 3000 endometrial cancer survivors. Another strength is the amount of medical record data. By having complete EMR data from 2 of the biggest medical care providers in the state of Utah as well as complete statewide ambulatory surgery and inpatient data, we were able to capture the baseline Charlson Comorbidity Index. We also had data on obesity at baseline through the UPDB. Most studies on cancer survival that are population-based have not been able to report on obesity. Also, through UPDB and UCR, we were able to have data from numerous sources to assess demographic and cancer specific risk factors in rural and urban areas.

Overall, many of the expected risk factors for death in endometrial cancer patients were significantly increased in both rural and urban areas. However, there were some significant differences including rural endometrial cancer patients in more advanced stages having higher risk of death. More research needs to be done specifically looking at rural endometrial cancer patients in advanced stages to learn what can be done to reduce the mortality. Future studies should also examine not only where patients live at diagnosis, but where they are receiving treatment and how that impacts their survival.

## Data Availability

The data that support the findings of this study are available from the Utah Population Database but restrictions apply to the availability of these data, which were used under license for the current study, and so are not publicly available. Data are however available from the authors upon reasonable request and with permission of the Utah Population Database.

## References

[1] Cancer Treatment & Survivorship Facts & Figures 2016-2017. In*.* Atlanta: American Cancer Society; 2016.

[2] Sheikh MA, Althouse AD, Freese KE, Soisson S, Edwards RP, Welburn S, Sukumvanich P, Comerci J, Kelley J, LaPorte RE (2014). USA endometrial cancer projections to 2030: should we be concerned?. Future Oncol (London).

[3] American Cancer Society. Cancer Facts & Figures 2019. Atlanta: American Cancer Society; 2019.

[4] Siegel RL, Miller KD, Jemal A (2016). Cancer statistics, 2016. CA Cancer J Clin.

[5] Rauh-Hain JA, Buskwofie A, Clemmer J, Boruta DM, Schorge JO, del Carmen MG (2015). Racial disparities in treatment of high-grade endometrial cancer in the Medicare population. Obstet Gynecol.

[6] Dessai SB, Adrash D, Geetha M, Arvind S, Bipin J, Nayanar S, Sachin K, Biji MS, Balasubramanian S (2016). Pattern of care in operable endometrial cancer treated at a rural-based tertiary care cancer center. Indian journal of cancer.

[7] Bregar Amy J., Alejandro Rauh-Hain J., Spencer Ryan, Clemmer Joel T., Schorge John O., Rice Laurel W., del Carmen Marcela G. (2017). Disparities in receipt of care for high-grade endometrial cancer: A National Cancer Data Base analysis. Gynecologic Oncology.

[8] Nicholas Z, Hu N, Ying J, Soisson P, Dodson M, Gaffney DK (2014). Impact of comorbid conditions on survival in endometrial cancer. Ame J Clin Oncol.

[9] Modesitt SC, Huang B, Shelton BJ, Wyatt S (2006). Endometrial cancer in Kentucky: the impact of age, smoking status, and rural residence. Gynecol Oncol.

[10] Board PDQATE (2002). Endometrial Cancer Treatment (PDQ(R)): Patient Version. PDQ Cancer Information Summaries.

[11] Zahnd WE, Hyon KS, Diaz-Sylvester P, Izadi SR, Colditz GA, Brard L (2018). Rural-urban differences in surgical treatment, regional lymph node examination, and survival in endometrial cancer patients. Cancer Causes Control.

[12] Overview - Utah Population Database - - Huntsman Cancer Institute - University of Utah Health Care - Salt Lake City, Utah. http://healthcare.utah.edu/huntsmancancerinstitute/research/updb/. Accessed 8 July 2019.

[13] Charlson ME, Pompei P, Ales KL, MacKenzie CR (1987). A new method of classifying prognostic comorbidity in longitudinal studies: development and validation. J Chronic Dis.

[14] Hadley Emily E., Discacciati Andrea, Costantine Maged M., Munn Mary B., Pacheco Luis D., Saade George R., Chiossi Giuseppe (2017). Maternal obesity is associated with chorioamnionitis and earlier indicated preterm delivery among expectantly managed women with preterm premature rupture of membranes. The Journal of Maternal-Fetal & Neonatal Medicine.

[15] Rural Urban Commuting Area Codes Data. http://depts.washington.edu/uwruca/ruca-urban.php. Accessed 8 July 2019.

[16] UDS Mapper. https://www.udsmapper.org. Accessed 8 July 2019.

[17] Felix AS, Weissfeld JL, Stone RA, Bowser R, Chivukula M, Edwards RP, Linkov F (2010). Factors associated with Type I and Type II endometrial cancer. Cancer Causes Control.

[18] Yang HP, Wentzensen N, Trabert B, Gierach GL, Felix AS, Gunter MJ, Hollenbeck A, Park Y, Sherman ME, Brinton LA (2013). Endometrial Cancer Risk Factors by 2 Main Histologic Subtypes: The NIH-AARP Diet and Health Study. Am J Epidemiol.

[19] Weaver KE, Geiger AM, Lu L, Case LD (2013). Rural-urban disparities in health status among US cancer survivors. Cancer.

[20] Schootman M, Homan S, Weaver KE, Jeffe DB, Yun S (2013). The health and welfare of rural and urban cancer survivors in Missouri. Prev Chronic Dis.

[21] Dejardin O, Bouvier AM, Faivre J, Boutreux S, De Pouvourville G, Launoy G (2008). Access to care, socioeconomic deprivation and colon cancer survival. Aliment Pharmacol Ther.

[22] Ruterbusch JJ, Ali-Fehmi R, Olson SH, Sealy-Jefferson S, Rybicki BA, Hensley-Alford S, Elshaikh MA, Gaba AR, Schultz D, Munkarah AR (2014). The influence of comorbid conditions on racial disparities in endometrial cancer survival. Am J Obstetr Gynecol.

[23] Cote ML, Ruterbusch JJ, Olson SH, Lu K, Ali-Fehmi R (2015). The Growing Burden of Endometrial Cancer: A Major Racial Disparity Affecting Black Women. Cancer Epidemiol Biomarkers Prev.

[24] Olson SH, Atoria CL, Cote ML, Cook LS, Rastogi R, Soslow RA, Brown CL, Elkin EB (2012). The impact of race and comorbidity on survival in endometrial cancer. Cancer Epidemiol Biomarkers Prev.

[25] Mahdi H, Schlick CJ, Kowk LL, Moslemi-Kebria M, Michener C (2014). Endometrial cancer in Asian and American Indian/Alaskan Native women: tumor characteristics, treatment and outcome compared to non-Hispanic white women. Gynecol Oncol.

